# Receptor‐Targeted Antiemetic Strategies in Cats: A Systematic Review and Intervention‐Specific Meta‐Analysis

**DOI:** 10.1002/vms3.71172

**Published:** 2026-08-14

**Authors:** Uğur Ersöz, Çağlar Özkalipci

**Affiliations:** ^1^ Faculty of Veterinary Medicine, Department of Surgery Atatürk University Erzurum Türkiye

**Keywords:** antiemetics, cats, emesis, maropitant, meta‐analysis, systematic review

## Abstract

**Background:**

Emesis is a common adverse effect in cats receiving alpha‐2 adrenergic agonists and opioids and may compromise perioperative welfare and safety. Comparative evidence for feline antiemetic protocols remains limited by heterogeneity in triggers, timing and outcomes.

**Objectives:**

To evaluate receptor‐targeted antiemetic strategies in cats and quantitatively synthesise controlled event‐level evidence for emesis incidence where appropriate.

**Methods:**

This systematic review followed PRISMA 2020 principles. Controlled feline studies assessing maropitant, ondansetron or metoclopramide for prevention or treatment of emesis were eligible. The primary meta‐analysis was restricted to maropitant versus control. Risk ratios (RRs) were pooled on the log scale using a random‐effects model with Paule–Mandel estimation and Hartung–Knapp adjustment.

**Results:**

Five controlled studies contributed to the primary maropitant analysis. Emesis occurred in 11/141 cats given maropitant and 70/152 controls. Maropitant reduced emesis risk (RR = 0.20, 95% confidence interval [CI]: 0.08–0.55), with low‐to‐moderate heterogeneity (*I* ^2^ = 23.2%; *Q* = 5.21, *p* = 0.27; tau = 0.37). Leave‐one‐out analyses consistently favoured maropitant. The approximate prediction interval was 0.04–1.05, indicating uncertainty about the magnitude of effect in future settings. Ondansetron evidence was limited to two evidence sources and suggested possible timing‐dependent benefit, whereas metoclopramide evidence was limited to one directly comparable binary study and was insufficient for pooled inference.

**Conclusions:**

Maropitant currently has the most consistent controlled evidence for reducing emesis incidence in cats under perioperative or induced‐emesis conditions. Further adequately powered, blinded and randomised trials using standardised vomiting, retching and nausea outcomes are needed.

## Graphical abstract

Maropitant showed the most consistent controlled evidence for reducing emesis incidence in cats under perioperative or induced‐emesis conditions. Ondansetron may be beneficial in timing‐specific settings, whereas metoclopramide evidence remains insufficient for pooled inference.

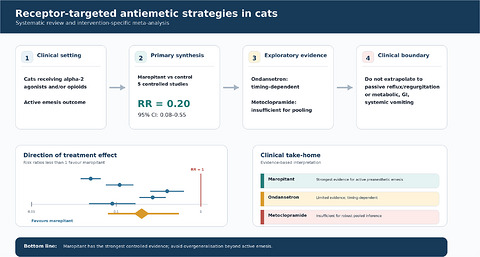

## 1 Introduction

Nausea and vomiting are related but distinct clinical phenomena. Vomiting is an observable motor reflex resulting in forceful expulsion of gastric contents, whereas nausea is a subjective aversive sensation that is difficult to measure directly in non‐verbal species (Trepanier 2010; Andrews et al. 2023). In cats, most studies therefore rely on observable surrogate signs such as salivation, lip licking, retching, altered posture, lethargy and restlessness. This distinction is important because an antiemetic intervention may reduce overt vomiting without consistently reducing nausea‐associated behaviours. Active vomiting should also be distinguished from passive gastroesophageal reflux or regurgitation during anaesthesia. Vomiting is an active reflex involving coordinated autonomic, respiratory and abdominal motor activity, whereas reflux or regurgitation may occur passively during induction, anaesthesia or muscle relaxation when protective airway reflexes are reduced. This distinction is clinically important because NK1 receptor antagonism may reduce centrally mediated active emesis, but it does not necessarily imply protection against passive retrograde movement of gastric contents, silent regurgitation or aspiration risk during the perioperative period.

Perioperative emesis is common in cats receiving alpha‐2 adrenergic agonists, opioids or combinations of both, particularly in experimental and perioperative models using dexmedetomidine and morphine (Santos et al. 2011; Martin‐Flores, Sakai, Learn, et al. 2016; Martin‐Flores, Sakai, Mastrocco, et al. 2016; Martin‐Flores et al. 2017). In addition to being unpleasant, vomiting may increase the risk of aspiration, esophagitis, delayed recovery, increases in intraocular or intracranial pressure and perioperative stress. These risks are particularly relevant in sedated or anesthetized cats, in which airway protection and physiologic reserve may be reduced.

The receptor pathways involved in feline emesis are species‐ and stimulus‐dependent. Neurokinin‐1 receptor signalling mediated by substance P is a major target for broad‐spectrum antiemetic therapy, and maropitant is the principal NK1 receptor antagonist used in veterinary practice (Hickman et al. 2008; Andrews et al. 2023). Ondansetron, a 5‐hydroxytryptamine type 3 receptor antagonist, is commonly used for nausea and vomiting, especially when serotonergic mechanisms are suspected (Santos et al. 2011; Kenward et al. 2017). Metoclopramide, a dopamine D2 receptor antagonist with prokinetic activity and 5‐HT3 antagonist activity at higher doses, has also been evaluated in cats, although its role in alpha‐2 agonist‐induced emesis is less clearly established (Kolahian and Jarolmasjed 2010; Trepanier 2010). A xylazine‐focused review has also summarized the diversity of antiemetic mechanisms evaluated in cats (Kolahian 2014).

Previous feline studies have suggested that maropitant may provide more consistent protection against emesis than ondansetron or metoclopramide under perioperative or experimentally induced emetic conditions (Martin‐Flores, Sakai, Learn, et al. 2016; Martin‐Flores, Sakai, Mastrocco, et al. 2016; Martin‐Flores et al. 2017; Gölgeli Bedir et al. 2025; Rocha et al. 2025). However, pooling all receptor‐targeted agents together would be statistically and biologically problematic because available studies differ in emetic trigger, timing of administration, comparator structure, outcome definition and availability of event‐level data. The present review therefore used an intervention‐specific approach. Accordingly, the quantitative conclusions of this review apply primarily to acute preanaesthetic or experimentally induced emesis associated with alpha‐2 adrenergic agonists and/or opioids, particularly dexmedetomidine and morphine models, and should not be extrapolated directly to vomiting caused by metabolic, gastrointestinal or systemic disease.

## 2 Materials and Methods

### 2.1 Study Design and Reporting

This study was designed as a systematic review with intervention‐specific quantitative synthesis. Reporting followed PRISMA 2020 principles for systematic reviews and meta‐analyses, including structured reporting of eligibility criteria, information sources, study selection, data extraction, risk‐of‐bias assessment and synthesis methods (Page et al. 2021). Because the objective was to produce a clinically interpretable and statistically defensible synthesis, quantitative pooling was performed only when studies were sufficiently comparable and provided extractable event‐level data.

A review protocol was developed before quantitative synthesis; however, the review was not formally preregistered in PROSPERO. During data extraction and evidence classification, clinical and methodological heterogeneity across interventions, emetic triggers, comparator structures and outcome formats became apparent. The final analysis plan was therefore refined before the final meta‐analysis to use an intervention‐specific approach: the primary pooled analysis was restricted to maropitant versus control studies with extractable binary emesis incidence data, ondansetron was treated as exploratory, and metoclopramide was synthesised narratively. These refinements affected the scope of quantitative synthesis but did not change the extracted event‐level data.

### 2.2 Search Strategy and Information Sources

Electronic searches were conducted in PubMed, Scopus and Web of Science for studies published from January 2008 to 15 May 2026. The final search update was completed on 15 May 2026. Search terms combined feline population terms with emesis‐related terms and antiemetic intervention terms. No restriction was applied according to study setting, emetic trigger or route of administration at the search stage.

The core search concept was (cat OR feline) AND (emesis OR vomiting OR nausea) AND (maropitant OR ondansetron OR metoclopramide). Database‐specific search strategies were adapted to the syntax of each database and are provided below to improve reproducibility.

No separate search was performed in veterinary‐specific, regional or specialist anaesthesia databases beyond PubMed, Scopus and Web of Science.

*PubMed search strategy*

((cat[Title/Abstract] OR cats[Title/Abstract] OR feline[Title/Abstract] OR felines[Title/Abstract]) AND (emesis[Title/Abstract] OR vomiting[Title/Abstract] OR vomit*[Title/Abstract] OR nausea[Title/Abstract] OR retching[Title/Abstract]) AND (maropitant[Title/Abstract] OR ondansetron[Title/Abstract] OR metoclopramide[Title/Abstract] OR antiemetic*[Title/Abstract] OR anti‐emetic*[Title/Abstract]))

*Scopus search strategy*

TITLE‐ABS‐KEY(cat OR cats OR feline OR felines) AND TITLE‐ABS‐KEY(emesis OR vomiting OR vomit* OR nausea OR retching) AND TITLE‐ABS‐KEY(maropitant OR ondansetron OR metoclopramide OR antiemetic* OR anti‐emetic*)

*Web of Science search strategy*

TS = (cat OR cats OR feline OR felines) AND TS = (emesis OR vomiting OR vomit* OR nausea OR retching) AND TS = (maropitant OR ondansetron OR metoclopramide OR antiemetic* OR anti‐emetic*)

Reference lists of included articles and relevant review articles were screened manually to identify additional eligible studies. Studies identified through the updated search or citation tracking were evaluated using the same eligibility criteria as database‐derived records.

### 2.3 Eligibility Criteria

Studies were eligible for qualitative synthesis if they evaluated domestic cats and assessed maropitant, ondansetron or metoclopramide for prevention or treatment of emesis, retching or nausea‐associated signs. Controlled randomised, blinded, experimental and clinical trials were eligible. Studies were excluded from quantitative synthesis if they were conducted in species other than cats, lacked a relevant control or comparator group or reported no extractable outcome data, defined for quantitative synthesis as group‐level binary emesis incidence data including the number of cats with at least one emetic event and the corresponding group denominator.

For the primary meta‐analysis, additional criteria were applied: studies had to compare maropitant with a control condition in cats and provide binary event‐level emesis incidence data. Ondansetron was considered for secondary exploratory quantitative synthesis only. Metoclopramide was not pooled because only one controlled feline study provided binary event‐level emesis incidence data suitable for risk‐ratio estimation. Studies evaluating eligible antiemetic drugs were excluded from quantitative synthesis when they lacked a relevant control group, used an active‐comparator‐only clinical design, reported only episode‐count outcomes without binary emesis incidence, or evaluated a route/formulation in an uncontrolled case‐description design.

### 2.4 Study Selection Process

After duplicate removal, titles and abstracts were screened against the eligibility criteria. Full texts were then assessed for final inclusion in the qualitative and quantitative syntheses. Study selection decisions were documented using the PRISMA 2020 flow structure. Reasons for exclusion at the full‐text stage were recorded, including non‐feline species, absence of a relevant control or comparator group, absence of extractable event‐level emesis data, review article design, case report design, conference abstract format without sufficient full‐text data, or outcome structure unsuitable for binary meta‐analysis.

Titles, abstracts and full‐text reports were screened by one reviewer. Data extraction and eligibility classification were also performed by one reviewer, with uncertain decisions reassessed during construction of the event‐level dataset and evidence classification table. No formal independent duplicate screening or duplicate data extraction was performed. This single‐reviewer process was treated as a major methodological limitation.

### 2.5 Outcomes

The primary outcome was emesis incidence, defined as the number of cats experiencing at least one vomiting event during the reported observation period. Secondary outcomes included retching, number of vomiting episodes, salivation, lip licking and other nausea‐associated behavioural signs when reported. Because nausea scores and behavioural definitions varied across studies, nausea‐associated outcomes were synthesised narratively rather than pooled.

### 2.6 Data Extraction and Study Classification

Extracted variables included author, year, study design, sample size, species, intervention, comparator, emetic trigger, dose, route, timing of administration, follow‐up period, emesis events, group denominators, retching and nausea‐associated signs. Each study was classified as eligible for the primary maropitant meta‐analysis, secondary ondansetron exploratory analysis, narrative synthesis only or exclusion from feline quantitative synthesis.

Multi‐arm studies were handled conservatively. In the primary maropitant analysis, each control group was used only once. For ondansetron, the Santos et al. trial included two ondansetron timing arms sharing the same control group; therefore, the two ondansetron arms were combined for the main secondary exploratory analysis to avoid double‐counting the control group. A timing‐focused sensitivity analysis was considered separately. For Gölgeli Bedir et al. (2025), the same saline control group contributed to drug‐specific comparisons involving maropitant, ondansetron and metoclopramide. These contrasts were therefore not treated as statistically independent evidence for comparative efficacy between drugs. No combined multidrug meta‐analysis, network meta‐analysis or formal indirect comparison was performed. The maropitant comparison contributed to the primary meta‐analysis, whereas ondansetron and metoclopramide findings were interpreted as exploratory or narrative evidence.

### 2.7 Risk of Bias and Certainty of Evidence

Risk of bias was assessed at the outcome level for studies included in the primary quantitative synthesis and for additional controlled or clinically relevant studies retained for exploratory or narrative synthesis. For the main text, risk‐of‐bias interpretation focused on the five studies contributing to the primary maropitant meta‐analysis. Table S2 provides the broader domain‐level assessment for all studies assessed for risk of bias, including studies retained for exploratory or narrative interpretation. Randomised controlled studies were evaluated using the Cochrane RoB 2 framework, which addresses bias arising from the randomisation process, deviations from intended interventions, missing outcome data, outcome measurement and selection of the reported result (Higgins et al. 2024). Non‐randomised, sequential‐dose or repeated‐measures studies included only in the narrative synthesis were evaluated using ROBINS‐I principles (Sterne et al. 2016). Domain‐level judgments and rationales are provided in Table S2. Certainty was rated as moderate rather than high because of imprecision related to the small number of studies, limited event counts and uncertainty around the stability of the estimated effect across future comparable clinical settings. The prediction interval was reported to contextualise expected between‐study variability and the expected range of effects in future comparable settings, but was not used as a standalone GRADE downgrading criterion.

### 2.8 Statistical Analysis

Risk ratios (RRs) with 95% confidence intervals (CIs) were calculated for each eligible controlled study. The primary quantitative synthesis was restricted to maropitant versus control and was performed on the natural logarithm of the RR. A random‐effects model was selected a priori because clinical and methodological variation was expected across studies, including differences in emetic trigger, route of administration, dosing interval and perioperative context.

Between‐study variance was estimated using the Paule–Mandel estimator. Hartung–Knapp adjustment was applied to the pooled random‐effects estimate to account for uncertainty associated with the small number of included studies. Heterogeneity was assessed using Cochran's *Q*, tau‐squared, tau and I‐squared.

A continuity correction of 0.5 was applied only to studies containing a zero cell. A risk‐ratio framework was retained because the primary outcome was cumulative emesis incidence and because RRs provide a clinically interpretable measure of relative risk for binary emesis outcomes. The continuity correction was applied only to the zero‐cell study to permit log‐risk‐ratio estimation while avoiding unnecessary modification of studies without zero cells. Alternative sparse‐data approaches, including Peto odds ratios or Mantel–Haenszel odds‐ratio methods, were not selected for the primary analysis because the outcome was not uniformly rare; treatment effects were expected to be large in some studies and clinical heterogeneity across emetic triggers, timing and routes of administration supported a random‐effects risk‐ratio framework. The influence of the zero‐cell study was evaluated through leave‐one‐out sensitivity analysis.

No continuity correction was applied to studies without zero cells. An approximate 95% prediction interval was calculated on the log risk‐ratio scale and back‐transformed to the RR scale to describe the expected range of effects in a future comparable study. Leave‐one‐out sensitivity analysis was performed by sequentially removing each study and recalculating the pooled estimate.

Ondansetron analyses were treated as exploratory because the available evidence was limited, timing‐dependent and affected by shared‐control structure. For the Santos et al. trial, the two ondansetron timing arms were combined for the main exploratory comparison to avoid double‐counting the shared control group. Metoclopramide was synthesised narratively because only one eligible controlled feline study provided binary event‐level emesis incidence data suitable for risk‐ratio estimation. Analyses were implemented using reproducible R code with the metafor package (Viechtbauer 2010) and the meta package.

## 3 Results

### 3.1 Study Selection and Evidence Classification

**FIGURE 1:**
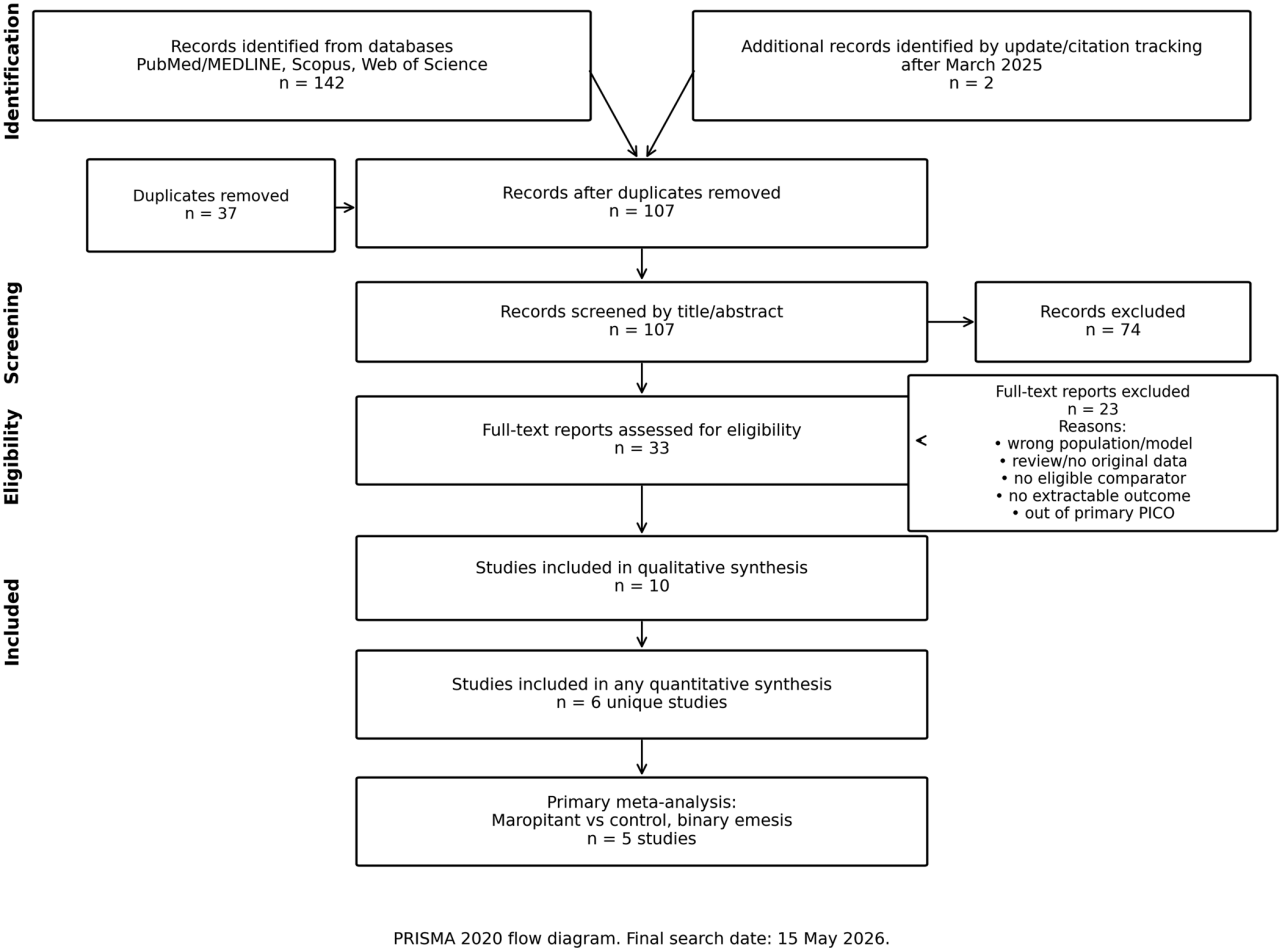
PRISMA 2020 flow diagram. The diagram summarizes record identification, duplicate removal, title and abstract screening, full‐text eligibility assessment, qualitative synthesis and quantitative synthesis. Reasons for full‐text exclusion are provided in Table S3. Studies discussed only as contextual narrative evidence and not included in controlled quantitative synthesis are distinguished from studies contributing event‐level data.

Database searches identified 142 records, and two additional records were identified through citation tracking or the updated search. After removal of 37 duplicates, 107 records were screened by title and abstract. Thirty‐three full‐text reports were assessed for eligibility. Ten studies were included in the qualitative synthesis, six unique studies contributed to any quantitative synthesis, and five studies contributed to the primary maropitant versus control meta‐analysis. Studies excluded from quantitative synthesis were excluded primarily because of non‐feline species, lack of a relevant control group, active‐comparator‐only design, absence of extractable binary emesis incidence data, episode‐count‐only outcomes or narrative/review design. The study selection process is summarised in Figure 1.

Particular attention was paid to recently published feline studies identified during the updated search. Gölgeli Bedir et al. and Rocha et al. met the criteria for inclusion in the quantitative maropitant synthesis because they provided controlled feline event‐level emesis incidence data. Baykurt Karata et al. (2025) was retained for narrative synthesis because it evaluated maropitant and ondansetron in cats with naturally occurring acute vomiting, but its active‐comparator design and clinical outcome structure did not match the primary prophylactic maropitant‐versus‐control comparison. Boukaache et al. (2022) was not included in the controlled synthesis because it was an uncontrolled case‐description study of transdermal maropitant and did not provide a relevant comparator group.

Ten studies were included in the qualitative synthesis. Of these, six unique studies contributed to at least one quantitative or exploratory event‐level comparison, and five studies contributed to the primary maropitant versus control meta‐analysis. Boukaache et al. (2022) was not included in any controlled quantitative synthesis because it was an uncontrolled case‐description study without a relevant comparator group; it was mentioned only as contextual narrative evidence regarding an alternative maropitant formulation. The revised evidence classification is presented in Table 1.

**TABLE 1:** Evidence classification for the revised synthesis.

Evidence category	Studies/examples	Role in revised manuscript
Primary quantitative synthesis	Martin‐Flores, Sakai, Learn et al. 2016; Martin‐Flores, Sakai, Mastrocco, et al. 2016; Martin‐Flores et al. 2017; Gölgeli Bedir et al. 2025; Rocha et al. 2025	Maropitant vs. control meta‐analysis of binary emesis incidence
Secondary exploratory synthesis	Santos et al. 2011; Gölgeli Bedir et al. 2025	Ondansetron vs. control; interpreted cautiously because of timing and shared‐control complexity
Narrative synthesis	Kolahian and Jarolmasjed 2010; Jarolmasjed and Kolahian 2010; Hickman et al. 2008; Baykurt Karata et al. 2025	Episode‐count outcomes, acute clinical vomiting, or models not suitable for the primary pooled binary analysis
Contextual narrative only; excluded from controlled quantitative synthesis	Boukaache et al. 2022	Uncontrolled case‐description design of transdermal maropitant; no relevant comparator group; not included in meta‐analysis
Background/mechanistic context	Trepanier 2010; Kenward et al. 2017; Andrews et al. 2023; Kolahian 2014; dog studies and reviews	Mechanistic or contextual interpretation only; not included in feline pooled effect estimates

### 3.2 Primary Meta‐Analysis: Maropitant Versus Control

Five controlled feline studies were eligible for the primary comparison of maropitant versus control. Across these studies, emesis occurred in 11 of 141 cats receiving maropitant and in 70 of 152 control cats. Individual study RRs consistently favoured maropitant. However, this consistency in direction should not be interpreted as statistical conclusiveness for every individual study, because at least one study‐level CI crossed the null value. The event‐level dataset for the primary maropitant analysis is presented in Table 2.

**TABLE 2:** Event‐level dataset for the primary maropitant meta‐analysis.

Study	Maropitant events/total	Control events/total	Trigger	Timing	Study RR
Martin‐Flores, Sakai, Learn, et al. 2016	1/32	20/34	Dexmedetomidine + morphine	20 h before	0.05
Martin‐Flores, Sakai, Mastrocco, et al. 2016	2/46	20/50	Dexmedetomidine + morphine	18 h before	0.11
Martin‐Flores et al. 2017	5/39	14/44	Dexmedetomidine + morphine	2–2.5 h before	0.40
Gölgeli Bedir et al. 2025	3/16	11/16	Dexmedetomidine	30 min before	0.27
Rocha et al. 2025	0/8	5/8	Dexmedetomidine + morphine	1 h before	0.09

Note: A continuity correction of 0.5 was applied for the Rocha et al. study because the maropitant arm contained zero emesis events. Continuity correction was not applied to studies without zero cells.

Random‐effects meta‐analysis showed that maropitant was associated with a substantial reduction in emesis risk compared with control conditions. The pooled RR was 0.20 (95% CI: 0.08–0.55), indicating an approximately 80% relative reduction in the risk of emesis. Between‐study heterogeneity was low to moderate and not statistically significant (I‐squared = 23.2%; *Q* = 5.21, *p* = 0.27; tau = 0.37).

**FIGURE 2:**
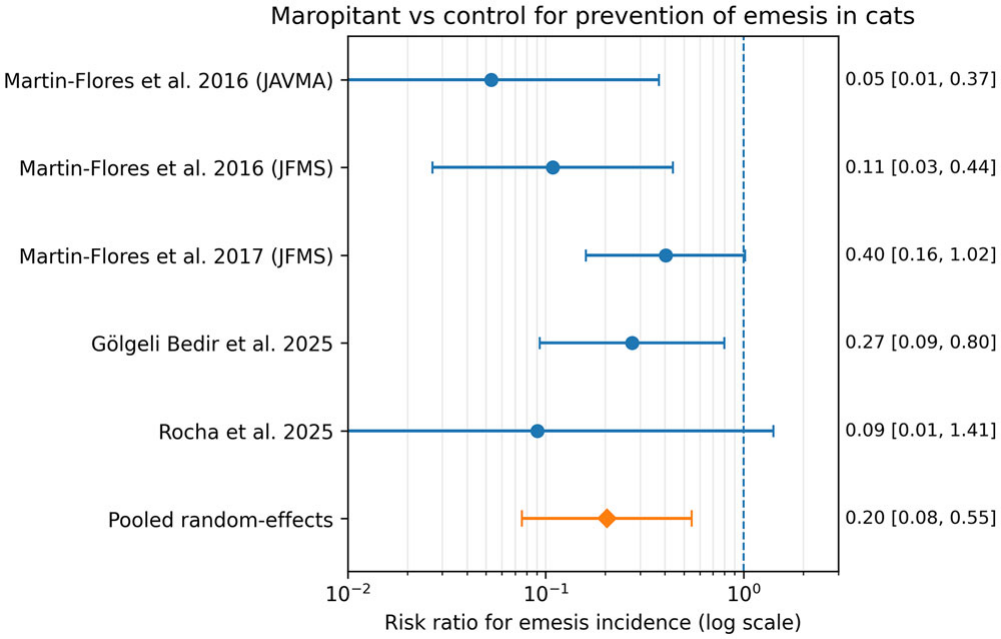
Forest plot for maropitant versus control. Risk ratios less than 1 favour maropitant. The pooled estimate was calculated using a random‐effects model with the Paule–Mandel estimator and Hartung–Knapp adjustment.

The approximate 95% prediction interval was 0.04–1.05. This wider interval indicates that, although the average pooled effect favoured maropitant, the magnitude of benefit expected in a future comparable study remains uncertain and may vary according to trigger, timing, route and clinical context. Therefore, the prediction interval was interpreted as evidence of residual uncertainty rather than as negation of the pooled treatment effect. Leave‐one‐out sensitivity analyses showed that the pooled effect remained directionally consistent after sequential removal of each study. The pooled RR was 0.26 after omitting Martin‐Flores, Sakai, Learn, et al. 2016, 0.23 after omitting Martin‐Flores, Sakai, Mastrocco, et al. 2016, 0.15 after omitting Martin‐Flores et al. 2017, 0.17 after omitting Gölgeli Bedir et al. 2025 and 0.20 after omitting Rocha et al. 2025. The corresponding forest plot is shown in Figure 2.

### 3.3 Secondary Exploratory Analysis: Ondansetron

Ondansetron evidence was more limited and timing‐dependent. Santos et al. evaluated two timing strategies using a shared control group: ondansetron administered 30 min before premedication and ondansetron mixed with premedication at the time of administration. When the two Santos ondansetron arms were combined to avoid double‐counting the shared control group, emesis occurred in 31/61 ondansetron‐treated cats and 22/28 controls (RR = 0.65, 95% CI: 0.47–0.89). Timing‐specific estimates suggested that ondansetron administered 30 min before premedication had a weaker effect (21/31 vs. 22/28; RR = 0.86, 95% CI: 0.63–1.18) than ondansetron administered with the premedication (10/30 vs. 22/28; RR = 0.42, 95% CI: 0.25–0.73). In Gölgeli Bedir et al., emesis occurred in 2/16 cats receiving ondansetron and 11/16 controls (RR = 0.18, 95% CI: 0.05–0.69). Because only two evidence sources were available and one involved shared‐control timing arms, these estimates were interpreted as exploratory rather than confirmatory pooled evidence.

When the two Santos ondansetron arms were combined to avoid double‐counting the shared control group and then considered together with Gölgeli Bedir et al., exploratory synthesis suggested a possible reduction in emesis risk. However, this estimate should not be interpreted as confirmatory because only two evidence sources were available, timing of administration differed between studies and the Hartung–Knapp CI was extremely imprecise. A selected‐timing sensitivity analysis using the Santos same‐time arm and Gölgeli Bedir et al. also favoured ondansetron, but this estimate remained unstable because *k* = 2. Ondansetron was therefore interpreted as potentially beneficial but low‐certainty and timing‐dependent.

### 3.4 Metoclopramide

A pooled binary meta‐analysis was not performed for metoclopramide because only one eligible controlled feline study provided extractable event‐level emesis incidence data. In Gölgeli Bedir et al., metoclopramide was associated with fewer vomiting events than saline control (2/16 vs. 11/16; study‐level RR = 0.18, 95% CI: 0.05–0.69). However, this estimate came from the same multi‐arm study and shared saline control group that also contributed to the maropitant and ondansetron drug‐specific comparisons. Therefore, it should be interpreted as a single study‐level contrast rather than as independent comparative evidence between antiemetic agents. Earlier metoclopramide work reported repeated‐measures episode‐count outcomes rather than directly comparable binary emesis incidence. The overall metoclopramide evidence was therefore considered insufficient for robust pooled inference.

### 3.5 Nausea‐Associated Signs and Retching

Nausea‐associated signs were reported inconsistently across studies. Maropitant generally reduced vomiting and retching, but effects on salivation and lip licking were less consistent. Ondansetron appeared to reduce nausea‐associated behavioural severity in some settings, particularly when administered with the emetogenic premedication, but outcome definitions varied across studies. Because nausea signs were measured using heterogeneous scales and observational criteria, these outcomes were not pooled.

### 3.6 Risk‐of‐Bias Assessment

The main‐text summary focuses on the five studies included in the primary maropitant meta‐analysis, whereas Table S2 provides the broader domain‐level assessment for studies included in exploratory or narrative synthesis. Domain‐level risk‐of‐bias judgments are presented in Table S2. For the five studies included in the primary maropitant meta‐analysis, the overall risk of bias for binary emesis incidence was judged as some concerns. These judgments were driven mainly by incomplete reporting of sequence generation or allocation concealment, limited availability of prospectively accessible protocols or statistical‐analysis plans and uncertainty around selective‐reporting safeguards. Missing outcome data and outcome measurement were generally judged as low risk for the primary binary emesis endpoint because denominators were extractable and emesis was an objective observable outcome. No study included in the primary maropitant meta‐analysis was judged to have high risk of bias for emesis incidence. Studies included only in the exploratory or narrative synthesis, particularly sequential‐dose or repeated‐measures studies, were judged to have greater methodological concern because of potential order, period, carryover and confounding effects.

## 4 Discussion

This systematic review and intervention‐specific meta‐analysis found that maropitant was consistently associated with a reduced incidence of emesis in controlled feline studies. Nevertheless, the individual study estimates varied in precision, and at least one study‐level CI crossed the null value; therefore, the conclusion rests on the pooled estimate and the consistent direction of effects rather than on uniformly conclusive individual trials. The pooled estimate indicated an approximately 80% relative reduction in emesis risk compared with control conditions. The effect direction was consistent across all included studies, heterogeneity was low to moderate, and leave‐one‐out sensitivity analyses showed that the pooled estimate remained directionally consistent after removal of each individual study. The recalculated pooled RRs ranged from 0.15, after omitting Martin‐Flores et al. (2017), to 0.26, after omitting Martin‐Flores, Sakai, Learn, et al. (2016), and all estimates continued to favour maropitant. This indicates that the primary finding was not driven by any single study. The strength of the maropitant evidence is supported by biological plausibility. Maropitant inhibits NK1 receptors and blocks substance P‐mediated signalling within central emetic pathways. In the included feline studies, emesis was most often triggered by alpha‐2 adrenergic agonists and/or opioids, which are clinically relevant perioperative emetic challenges. The consistency of benefit across subcutaneous and oral administration protocols suggests that NK1 receptor antagonism is a robust strategy for reducing overt vomiting in cats. Uncontrolled reports of alternative formulations, such as transdermal maropitant, may be clinically interesting but were outside the scope of the controlled intervention‐specific synthesis.

The prediction interval was wider than the CI and crossed the line of no effect. The CI and prediction interval answer related but distinct questions. The CI summarizes uncertainty around the mean pooled effect, whereas the prediction interval reflects the range of effects that might be expected across future comparable settings. The prediction interval crossing the line of no effect therefore supports a cautious interpretation of generalisability, particularly because only five studies were available and clinical context varied across trials. This does not negate the pooled benefit, but it indicates that the exact magnitude of benefit in a future trial remains uncertain. This is expected when only five studies are available and when triggers, dosing intervals and clinical contexts vary. From a clinically oriented evidence‐synthesis perspective, maropitant had the most consistent controlled evidence for reducing active emesis in cats exposed to alpha‐2 adrenergic agonist and/or opioid premedication, whereas ondansetron and metoclopramide findings remained exploratory because of limited evidence, timing heterogeneity and shared‐control structures.

Ondansetron findings require more cautious interpretation. The Santos et al. trial suggested that timing may be critical, with administration at the time of premedication appearing more effective than administration 30 min earlier. Gölgeli Bedir et al. also found fewer vomiting events with ondansetron than saline. However, the small number of eligible evidence sources, timing heterogeneity and shared‐control structure preclude strong pooled inference. Ondansetron may have a clinically meaningful role, especially for nausea‐associated signs, but the certainty of evidence is lower than for maropitant. The active‐comparator clinical study by Baykurt Karata et al. provides useful contextual evidence for naturally occurring acute vomiting in cats. However, because cats were randomised to maropitant or ondansetron without a placebo or untreated control group, the study does not estimate the absolute effect of either drug versus control and was therefore not eligible for the primary prophylactic maropitant‐versus‐control meta‐analysis. Its findings are best interpreted as supportive clinical context rather than direct evidence for the pooled estimate. The scope of the pooled evidence should be interpreted carefully. The primary maropitant meta‐analysis was dominated by studies evaluating acute emesis induced by dexmedetomidine, morphine or their combination in preanesthetic or perioperative settings. Therefore, the pooled estimate should not be interpreted as evidence of general superiority of maropitant for all feline vomiting syndromes. Vomiting associated with metabolic disease, gastrointestinal obstruction or inflammation, hepatic disease, uraemia or other systemic disorders may involve different mechanisms and clinical priorities, and these settings were not directly represented in the primary pooled analysis.

Evidence for metoclopramide was insufficient for pooled binary analysis. Although Gölgeli Bedir et al. reported a lower incidence of vomiting with metoclopramide compared with saline, earlier feline work used episode‐count outcomes in small repeated‐measures designs. These designs are clinically informative but cannot be combined directly with parallel‐group binary event data without additional assumptions. Therefore, metoclopramide should be described as having limited and low‐certainty evidence rather than as equivalent to maropitant or ondansetron. A plausible pharmacodynamic explanation for the limited and less consistent metoclopramide evidence is receptor‐pathway mismatch in the available feline models. Dexmedetomidine‐ and xylazine‐induced emesis is closely linked to alpha‐2 adrenergic mechanisms, whereas metoclopramide acts primarily as a dopamine D2 receptor antagonist with prokinetic properties and additional serotonergic activity at higher doses. Therefore, when emesis is predominantly triggered through alpha‐2 adrenergic pathways, D2 antagonism alone may be biologically less well matched to the emetic stimulus than NK1 receptor antagonism. This interpretation should remain cautious because the available feline metoclopramide evidence is sparse and not directly comparable across outcome formats.

An important methodological implication of this review is that receptor‐targeted antiemetic studies in cats should not be pooled indiscriminately across drugs, triggers and outcome types. Combining acute clinical vomiting, motion sickness, xylazine‐induced emesis, dexmedetomidine‐induced emesis and opioid‐associated perioperative emesis in a single pooled comparison would create a biologically and statistically heterogeneous estimate. The intervention‐specific approach used here preserves interpretability and reduces the risk of overclaiming comparative efficacy.

This review has limitations. The number of eligible controlled feline studies was small, and several outcomes were reported inconsistently. Dosing, timing, comparator conditions and emetic triggers varied across studies. Nausea‐associated signs were not standardised and are inherently difficult to validate in cats. Publication bias could not be formally assessed because fewer than 10 studies were available for the primary meta‐analysis. Risk‐of‐bias assessment indicated that the primary maropitant evidence base was not dominated by high‐risk studies; however, all primary studies had at least some concerns, mainly because allocation concealment, sequence‐generation details, protocol availability or selective‐reporting safeguards were not fully verifiable from the available reports. Sequential‐dose studies were interpreted narratively because their design increased susceptibility to order and carryover effects. The search strategy was limited to PubMed, Scopus and Web of Science. Although reference lists and relevant reviews were screened manually, the absence of separate searches in veterinary‐specific, regional or specialist anaesthesia databases may have reduced comprehensiveness and may have increased the risk of missing small or regionally indexed feline antiemetic studies. A major limitation of the review process was that study screening, eligibility assessment and data extraction were conducted by a single reviewer rather than by two independent reviewers. This may have increased the risk of selection error, omission of relevant studies and data‐extraction error, particularly for small feline antiemetic studies published in regional or specialist veterinary anaesthesia journals. Another limitation is the structural dependency introduced by shared control groups in multi‐arm studies. In particular, the same saline control group from Gölgeli Bedir et al. (2025) contributed to drug‐specific comparisons for maropitant, ondansetron and metoclopramide. Therefore, these drug‐specific findings should not be interpreted as independent comparative efficacy estimates between antiemetic agents.

Future feline trials should use adequately powered randomised blinded designs, standardised definitions of vomiting and retching, validated or clearly defined nausea behaviour scales, consistent observation windows and complete event‐level reporting. Trials directly comparing maropitant, ondansetron and metoclopramide under the same emetic trigger and timing conditions would be especially valuable. Additional outcomes such as gastroesophageal reflux, aspiration risk, recovery quality and perioperative pain should also be considered. Although the search was updated to 15 May 2026 and included three major bibliographic databases with citation tracking, the review was not prospectively registered, and the small size of the feline antiemetic literature limits the ability to fully assess selective publication or reporting patterns. Future perioperative trials should distinguish active vomiting and retching from passive reflux or regurgitation and should consider clinically relevant outcomes such as gastroesophageal reflux, silent regurgitation and aspiration‐related complications.

## 5 Certainty of Evidence

The certainty of evidence was highest for maropitant because five controlled feline studies provided directionally consistent event‐level binary emesis data and the pooled estimate showed a clear reduction in emesis incidence. Certainty was rated as moderate rather than high because of imprecision related to the small number of studies, limited event counts and uncertainty around the stability of the estimated effect across future comparable clinical settings. The prediction interval was reported to contextualise expected between‐study variability, but was not used as a standalone GRADE downgrading criterion. The evidence was not downgraded for inconsistency because all study‐level estimates favoured maropitant and statistical heterogeneity was low to moderate. The GRADE‐style certainty assessment is summarised in Table 3.

**TABLE 3:** GRADE‐style certainty assessment.

Outcome	Comparison	No. of studies	Main finding	Certainty	Rationale
Emesis incidence	Maropitant vs. control	5	RR = 0.20 (95% CI: 0.08–0.55)	Moderate	Downgraded one level for imprecision because the number of studies and events was limited and the precision of the estimated effect across future comparable settings remained uncertain. Not downgraded for inconsistency because all study‐level point estimates favoured maropitant and statistical heterogeneity was low to moderate. The prediction interval was interpreted as contextual information on expected between‐study variability rather than as a standalone GRADE downgrading criterion.
Emesis incidence	Ondansetron vs. control	Two evidence sources	Possible reduction in emesis risk, but pooled inference unstable	Low	Downgraded for serious imprecision and indirectness due to timing heterogeneity, limited number of evidence sources and shared‐control handling.
Emesis incidence	Metoclopramide vs. control	1 binary study	Insufficient for pooled inference	Very low	Downgraded for very serious imprecision and limited directly comparable binary outcome data.

Ondansetron evidence was rated as low certainty because only two evidence sources were available, timing of administration differed across studies, one trial involved shared‐control timing arms and pooled inference was highly imprecise.

Metoclopramide evidence was rated as very low certainty because only one eligible controlled feline study provided extractable binary emesis incidence data suitable for risk‐ratio estimation, while earlier studies used outcome structures that were not directly compatible with the primary binary meta‐analysis.

## 6 Conclusion

For cats at risk of alpha‐2 agonist‐ and/or opioid‐associated active preanesthetic emesis, maropitant has the strongest controlled evidence for reducing vomiting. Ondansetron evidence is limited and timing‐dependent, whereas metoclopramide evidence is insufficient for robust pooled inference in this setting. These findings should not be extrapolated to passive reflux, regurgitation or vomiting caused by metabolic, gastrointestinal or systemic disease.

## Author Contributions

**Uğur Ersöz**: conceptualisation, methodology, investigation, data curation, formal analysis, writing – original draft, writing – review and editing, supervision, funding acquisition, visualization, validation, software, project administration. **Çağlar Özkalipci**: validation, investigation, data curation, methodology, writing – review and editing.

## Conflicts of Interest

The authors declare no conflicts of interest.

## Funding

This study was financially supported by the Scientific Research Projects Coordination Unit of Atatürk University (BAP) under project ID 8846.

## Ethics Statement

The authors confirm that the ethical policies of the journal have been adhered to. No ethical approval was required because this systematic review and meta‐analysis used only published aggregate data and did not involve original animal or human participant research.

## Supporting information




**Supporting File 1**: vms371172‐sup‐0001‐SuppMat.docx

**Supporting File 2**: vms371172‐sup‐0002‐Dataset.csv

**Supporting File 3**: vms371172‐sup‐0003‐SuppMat.R

**Supporting File 4**: vms371172‐sup‐0004‐SuppMat.docx

## Data Availability

The data supporting the findings of this study are available in the Supporting Information of this article. The extracted aggregate dataset and reproducible R script are provided as Supporting Information Files (File S1: Reproducible R script for the primary meta‐analysis; File S2: Event‐level dataset). No individual animal‐level data were used.
