# Supplementary material for: Receptor‐Targeted Antiemetic Strategies in Cats: A Systematic Review and Intervention‐Specific Meta‐Analysis
Source: Vet Med Sci. 2026 Aug 14;12(5):e71172. doi: 10.1002/vms3.71172 (PMC13475627; doi:10.1002/vms3.71172)
Supplement: Supplementary file 1 [file VMS3-12-e71172-s002.docx]

**Supporting Information**

Receptor-Targeted Antiemetic Strategies in Cats: A Systematic Review and Intervention-Specific Meta-Analysis

# Table S1. Event-level dataset for the primary maropitant meta-analysis

Risk ratios less than 1 favour maropitant. The continuity correction of 0.5 was applied only to studies containing a zero cell.

| **Study** | **Maropitant events/total** | **Control events/total** | **Trigger** | **Timing** | **Study RR** |
| --- | --- | --- | --- | --- | --- |
| Martin-Flores et al. 2016 (JAVMA) | 1/32 | 20/34 | Dexmedetomidine + morphine | 20 h before | 0.05 |
| Martin-Flores et al. 2016 (JFMS) | 2/46 | 20/50 | Dexmedetomidine + morphine | 18 h before | 0.11 |
| Martin-Flores et al. 2017 (JFMS) | 5/39 | 14/44 | Dexmedetomidine + morphine | 2-2.5 h before | 0.40 |
| Gölgeli Bedir et al. 2025 | 3/16 | 11/16 | Dexmedetomidine | 30 min before | 0.27 |
| Rocha et al. 2025 | 0/8 | 5/8 | Dexmedetomidine + morphine | 1 h before | 0.09* |

# Table S2. Domain-level risk-of-bias assessment

RoB 2 domains: D1, randomisation process; D2, deviations from intended interventions; D3, missing outcome data; D4, measurement of outcome; D5, selection of reported result. ROBINS-I domains: D1, confounding; D2, selection of participants; D3, classification of interventions; D4, deviations from intended interventions; D5, missing data; D6, measurement of outcomes; D7, selection of reported result. Judgements apply to emesis incidence where event-level data were available and to the relevant narrative outcome when binary incidence was not available.

| **Study** | **Tool** | **Domain** | **Judgment** | **Overall** | **Rationale** |
| --- | --- | --- | --- | --- | --- |
| Martin-Flores et al. 2016 (JAVMA) | RoB 2 | D1: Randomisation process | Some concerns | Some concerns | Randomized design reported; allocation concealment or sequence-generation details not fully verifiable in the available text. |
| Martin-Flores et al. 2016 (JAVMA) | RoB 2 | D2: Deviations from intended interventions | Low | Some concerns | No important deviations from intended intervention were apparent for binary emesis. |
| Martin-Flores et al. 2016 (JAVMA) | RoB 2 | D3: Missing outcome data | Low | Some concerns | Denominators and binary emesis outcomes were extractable; missing outcome data did not appear to drive the result. |
| Martin-Flores et al. 2016 (JAVMA) | RoB 2 | D4: Measurement of outcome | Low | Some concerns | Binary emesis was directly observed and objectively defined. |
| Martin-Flores et al. 2016 (JAVMA) | RoB 2 | D5: Selection of reported result | Some concerns | Some concerns | Prospective protocol or statistical-analysis plan was not fully verifiable; selective-reporting risk cannot be excluded. |
| Martin-Flores et al. 2016 (JAVMA) | RoB 2 | Overall judgment | Some concerns | Some concerns | Randomized controlled trial; objective emesis outcome and blinded assessment are strengths. Allocation concealment and prospective protocol/analysis plan were not fully verifiable from the available manuscript. |
| Martin-Flores et al. 2016 (JFMS) | RoB 2 | D1: Randomisation process | Some concerns | Some concerns | Randomized design reported; allocation concealment or sequence-generation details not fully verifiable in the available text. |
| Martin-Flores et al. 2016 (JFMS) | RoB 2 | D2: Deviations from intended interventions | Some concerns | Some concerns | Comparator/timing or no-treatment design may introduce performance-related concerns. |
| Martin-Flores et al. 2016 (JFMS) | RoB 2 | D3: Missing outcome data | Low | Some concerns | Denominators and binary emesis outcomes were extractable; missing outcome data did not appear to drive the result. |
| Martin-Flores et al. 2016 (JFMS) | RoB 2 | D4: Measurement of outcome | Low | Some concerns | Binary emesis was directly observed and objectively defined. |
| Martin-Flores et al. 2016 (JFMS) | RoB 2 | D5: Selection of reported result | Some concerns | Some concerns | Prospective protocol or statistical-analysis plan was not fully verifiable; selective-reporting risk cannot be excluded. |
| Martin-Flores et al. 2016 (JFMS) | RoB 2 | Overall judgment | Some concerns | Some concerns | Prospective blinded randomized controlled design. No-treatment comparator rather than placebo may introduce performance-related concerns; protocol availability unclear. |
| Martin-Flores et al. 2017 (JFMS) | RoB 2 | D1: Randomisation process | Some concerns | Some concerns | Randomized design reported; allocation concealment or sequence-generation details not fully verifiable in the available text. |
| Martin-Flores et al. 2017 (JFMS) | RoB 2 | D2: Deviations from intended interventions | Some concerns | Some concerns | Comparator/timing or no-treatment design may introduce performance-related concerns. |
| Martin-Flores et al. 2017 (JFMS) | RoB 2 | D3: Missing outcome data | Low | Some concerns | Denominators and binary emesis outcomes were extractable; missing outcome data did not appear to drive the result. |
| Martin-Flores et al. 2017 (JFMS) | RoB 2 | D4: Measurement of outcome | Some concerns | Some concerns | Emesis is an objective observable outcome; nausea signs were less standardized when applicable. |
| Martin-Flores et al. 2017 (JFMS) | RoB 2 | D5: Selection of reported result | Some concerns | Some concerns | Prospective protocol or statistical-analysis plan was not fully verifiable; selective-reporting risk cannot be excluded. |
| Martin-Flores et al. 2017 (JFMS) | RoB 2 | Overall judgment | Some concerns | Some concerns | Randomized design with extractable binary emesis data. No-treatment control and limited clarity around blinding/allocation concealment introduce concerns, especially for nausea-associated signs. |
| Santos et al. 2011 | RoB 2 | D1: Randomisation process | Some concerns | Some concerns | Randomized design reported; allocation concealment or sequence-generation details not fully verifiable in the available text. |
| Santos et al. 2011 | RoB 2 | D2: Deviations from intended interventions | Low | Some concerns | No important deviations from intended intervention were apparent for binary emesis. |
| Santos et al. 2011 | RoB 2 | D3: Missing outcome data | Low | Some concerns | Denominators and binary emesis outcomes were extractable; missing outcome data did not appear to drive the result. |
| Santos et al. 2011 | RoB 2 | D4: Measurement of outcome | Low | Some concerns | Binary emesis was directly observed and objectively defined. |
| Santos et al. 2011 | RoB 2 | D5: Selection of reported result | Some concerns | Some concerns | Prospective protocol or statistical-analysis plan was not fully verifiable; selective-reporting risk cannot be excluded. |
| Santos et al. 2011 | RoB 2 | Overall judgment | Some concerns | Some concerns | Randomized, blinded, controlled trial. Shared control across two ondansetron timing arms was handled analytically; allocation concealment/protocol availability not fully verifiable. |
| Gölgeli Bedir et al. 2025 | RoB 2 | D1: Randomisation process | Some concerns | Some concerns | Randomized design reported; allocation concealment or sequence-generation details not fully verifiable in the available text. |
| Gölgeli Bedir et al. 2025 | RoB 2 | D2: Deviations from intended interventions | Some concerns | Some concerns | Comparator/timing or no-treatment design may introduce performance-related concerns. |
| Gölgeli Bedir et al. 2025 | RoB 2 | D3: Missing outcome data | Low | Some concerns | Denominators and binary emesis outcomes were extractable; missing outcome data did not appear to drive the result. |
| Gölgeli Bedir et al. 2025 | RoB 2 | D4: Measurement of outcome | Low | Some concerns | Binary emesis was directly observed and objectively defined. |
| Gölgeli Bedir et al. 2025 | RoB 2 | D5: Selection of reported result | Some concerns | Some concerns | Prospective protocol or statistical-analysis plan was not fully verifiable; selective-reporting risk cannot be excluded. |
| Gölgeli Bedir et al. 2025 | RoB 2 | Overall judgment | Some concerns | Some concerns | Randomized multi-arm controlled clinical trial with objective emesis/retching outcomes. Blinding/allocation concealment and prospective selective-reporting safeguards were not fully verifiable. |
| Baykurt Karataş et al. 2025 | RoB 2 | D1: Randomisation process | Some concerns | Some concerns | Randomized design reported; allocation concealment or sequence-generation details not fully verifiable in the available text. |
| Baykurt Karataş et al. 2025 | RoB 2 | D2: Deviations from intended interventions | Some concerns | Some concerns | Comparator/timing or no-treatment design may introduce performance-related concerns. |
| Baykurt Karataş et al. 2025 | RoB 2 | D3: Missing outcome data | Low | Some concerns | Denominators and binary emesis outcomes were extractable; missing outcome data did not appear to drive the result. |
| Baykurt Karataş et al. 2025 | RoB 2 | D4: Measurement of outcome | Some concerns | Some concerns | Emesis is an objective observable outcome; nausea signs were less standardized when applicable. |
| Baykurt Karataş et al. 2025 | RoB 2 | D5: Selection of reported result | Some concerns | Some concerns | Prospective protocol or statistical-analysis plan was not fully verifiable; selective-reporting risk cannot be excluded. |
| Baykurt Karataş et al. 2025 | RoB 2 | Overall judgment | Some concerns | Some concerns | Randomized active-comparator clinical study in naturally occurring acute vomiting. No placebo/control arm for primary prophylactic meta-analysis; subjective scoring outcomes create measurement concerns. |
| Rocha et al. 2025 | RoB 2 | D1: Randomisation process | Some concerns | Some concerns | Randomized design reported; allocation concealment or sequence-generation details not fully verifiable in the available text. |
| Rocha et al. 2025 | RoB 2 | D2: Deviations from intended interventions | Low | Some concerns | No important deviations from intended intervention were apparent for binary emesis. |
| Rocha et al. 2025 | RoB 2 | D3: Missing outcome data | Low | Some concerns | Denominators and binary emesis outcomes were extractable; missing outcome data did not appear to drive the result. |
| Rocha et al. 2025 | RoB 2 | D4: Measurement of outcome | Low | Some concerns | Binary emesis was directly observed and objectively defined. |
| Rocha et al. 2025 | RoB 2 | D5: Selection of reported result | Some concerns | Some concerns | Prospective protocol or statistical-analysis plan was not fully verifiable; selective-reporting risk cannot be excluded. |
| Rocha et al. 2025 | RoB 2 | Overall judgment | Some concerns | Some concerns | Double-blinded randomized parallel-group trial. Objective emesis incidence was extractable; small sample affects precision rather than domain-level RoB. Protocol/analysis-plan availability unclear. |
| Hickman et al. 2008 | ROBINS-I / experimental controlled model | D1: Confounding | Moderate | Moderate | Sequential/non-randomized or experimental structure creates susceptibility to confounding, order or period effects. |
| Hickman et al. 2008 | ROBINS-I / experimental controlled model | D2: Selection of participants | Moderate | Moderate | Participant selection was constrained by experimental/clinical setting; representativeness may be limited. |
| Hickman et al. 2008 | ROBINS-I / experimental controlled model | D3: Classification of interventions | Low | Moderate | Intervention classification was clear from dose, route and timing descriptions. |
| Hickman et al. 2008 | ROBINS-I / experimental controlled model | D4: Deviations from intended interventions | Moderate | Moderate | Potential deviations from intended interventions were not fully controlled or verifiable. |
| Hickman et al. 2008 | ROBINS-I / experimental controlled model | D5: Missing data | Low | Moderate | Missing data did not appear to be the main limitation based on available reporting. |
| Hickman et al. 2008 | ROBINS-I / experimental controlled model | D6: Measurement of outcomes | Low | Moderate | Outcome measurement relied on observed emesis/episode counts or clinical scoring; some subjective components were present. |
| Hickman et al. 2008 | ROBINS-I / experimental controlled model | D7: Selection of reported result | Some concerns | Moderate | Protocol availability was limited; selective reporting could not be fully excluded. |
| Hickman et al. 2008 | ROBINS-I / experimental controlled model | Overall judgment | Moderate | Moderate | Controlled experimental efficacy studies with objective emesis counts. Randomisation/concealment/blinding details and multiplicity across models were incompletely verifiable. |
| Kolahian & Jarolmasjed 2010 | ROBINS-I / sequential repeated-measures | D1: Confounding | Serious | Serious | Sequential/non-randomized or experimental structure creates susceptibility to confounding, order or period effects. |
| Kolahian & Jarolmasjed 2010 | ROBINS-I / sequential repeated-measures | D2: Selection of participants | Moderate | Serious | Participant selection was constrained by experimental/clinical setting; representativeness may be limited. |
| Kolahian & Jarolmasjed 2010 | ROBINS-I / sequential repeated-measures | D3: Classification of interventions | Low | Serious | Intervention classification was clear from dose, route and timing descriptions. |
| Kolahian & Jarolmasjed 2010 | ROBINS-I / sequential repeated-measures | D4: Deviations from intended interventions | Serious | Serious | Sequential or repeated-measures design may introduce period, order, carryover or learning/sedation effects. |
| Kolahian & Jarolmasjed 2010 | ROBINS-I / sequential repeated-measures | D5: Missing data | Low | Serious | Missing data did not appear to be the main limitation based on available reporting. |
| Kolahian & Jarolmasjed 2010 | ROBINS-I / sequential repeated-measures | D6: Measurement of outcomes | Some concerns | Serious | Outcome measurement relied on observed emesis/episode counts or clinical scoring; some subjective components were present. |
| Kolahian & Jarolmasjed 2010 | ROBINS-I / sequential repeated-measures | D7: Selection of reported result | Some concerns | Serious | Protocol availability was limited; selective reporting could not be fully excluded. |
| Kolahian & Jarolmasjed 2010 | ROBINS-I / sequential repeated-measures | Overall judgment | Serious | Serious | Sequential increasing-dose design without randomisation of dose order; possible period, order, carryover, and learning/sedation effects. Episode-count rather than binary event data. |
| Jarolmasjed & Kolahian 2010 | ROBINS-I / sequential repeated-measures | D1: Confounding | Serious | Serious | Sequential/non-randomized or experimental structure creates susceptibility to confounding, order or period effects. |
| Jarolmasjed & Kolahian 2010 | ROBINS-I / sequential repeated-measures | D2: Selection of participants | Moderate | Serious | Participant selection was constrained by experimental/clinical setting; representativeness may be limited. |
| Jarolmasjed & Kolahian 2010 | ROBINS-I / sequential repeated-measures | D3: Classification of interventions | Low | Serious | Intervention classification was clear from dose, route and timing descriptions. |
| Jarolmasjed & Kolahian 2010 | ROBINS-I / sequential repeated-measures | D4: Deviations from intended interventions | Serious | Serious | Sequential or repeated-measures design may introduce period, order, carryover or learning/sedation effects. |
| Jarolmasjed & Kolahian 2010 | ROBINS-I / sequential repeated-measures | D5: Missing data | Low | Serious | Missing data did not appear to be the main limitation based on available reporting. |
| Jarolmasjed & Kolahian 2010 | ROBINS-I / sequential repeated-measures | D6: Measurement of outcomes | Some concerns | Serious | Outcome measurement relied on observed emesis/episode counts or clinical scoring; some subjective components were present. |
| Jarolmasjed & Kolahian 2010 | ROBINS-I / sequential repeated-measures | D7: Selection of reported result | Some concerns | Serious | Protocol availability was limited; selective reporting could not be fully excluded. |
| Jarolmasjed & Kolahian 2010 | ROBINS-I / sequential repeated-measures | Overall judgment | Serious | Serious | Sequential increasing-dose design without randomisation of dose order; possible period, order, carryover, and learning/sedation effects. Episode-count rather than binary event data. |

# Table S3. Full-text exclusion reason categories

The table summarises the exclusion categories applied during full-text screening. Exact record-level decisions should be retained in the authors' screening log.

| **Reason category** | **Description** |
| --- | --- |
| Non-feline species | Reports not conducted in domestic cats. |
| No relevant control or comparator | Studies without a comparator suitable for controlled synthesis. |
| Active-comparator-only design | Clinical designs comparing active drugs without a placebo, untreated or saline control; retained narratively where relevant. |
| No extractable binary event-level emesis data | Reports without group-level emesis events and denominators suitable for risk-ratio estimation. |
| Episode-count-only outcomes | Studies reporting numbers of episodes rather than numbers of cats with at least one emetic event. |
| Review, narrative or case-report design | Articles not providing controlled primary event-level data. |
| Conference abstract or insufficient full text | Reports with insufficient methodological or outcome detail for synthesis. |

Additional Supporting Information files

File S1. Reproducible R script for the primary meta-analysis.

File S2. Event-level dataset for the primary meta-analysis.

File S3. PRISMA 2020 checklist.
