# Supplementary material for: Receptor‐Targeted Antiemetic Strategies in Cats: A Systematic Review and Intervention‐Specific Meta‐Analysis
Source: Vet Med Sci. 2026 Aug 14;12(5):e71172. doi: 10.1002/vms3.71172 (PMC13475627; doi:10.1002/vms3.71172)
Supplement: Supplementary file 2 [file VMS3-12-e71172-s004.docx]

**PRISMA 2020 Checklist**

Receptor-Targeted Antiemetic Strategies in Cats: A Systematic Review and Intervention-Specific Meta-Analysis

This checklist is prepared for submission and maps PRISMA 2020 items to the revised manuscript sections.

| **Section/topic** | **Item** | **Checklist item** | **Location in manuscript** |
| --- | --- | --- | --- |
| Title | 1 | Identify the report as a systematic review. | Title |
| Abstract | 2 | Provide a structured summary including background, objectives, methods, results, limitations and conclusions. | Abstract |
| Introduction | 3 | Describe the rationale for the review in the context of existing knowledge. | Introduction |
| Introduction | 4 | Provide an explicit statement of the objectives or questions addressed. | Abstract; Introduction |
| Methods | 5 | Specify eligibility criteria and how studies were grouped for synthesis. | Materials and Methods, Eligibility criteria |
| Methods | 6 | Specify all information sources and the date when each source was last searched. | Materials and Methods, Search strategy and information sources |
| Methods | 7 | Present the full search strategies for all databases. | Materials and Methods, Search strategy and information sources |
| Methods | 8 | Specify the methods used to decide whether each study met inclusion criteria. | Materials and Methods, Study selection process |
| Methods | 9 | Specify the methods used to collect data from reports. | Materials and Methods, Data extraction and study classification |
| Methods | 10 | List and define all outcomes and other variables for which data were sought. | Materials and Methods, Outcomes; Data extraction and study classification |
| Methods | 11 | Specify methods used to assess risk of bias in included studies. | Materials and Methods, Risk of bias and certainty of evidence |
| Methods | 12 | Specify effect measures for each outcome. | Materials and Methods, Statistical analysis |
| Methods | 13 | Describe methods used to synthesise results. | Materials and Methods, Statistical analysis |
| Methods | 14 | Describe methods used to assess risk of reporting bias. | Materials and Methods, Risk of bias and certainty of evidence |
| Methods | 15 | Describe methods used to assess certainty of evidence. | Materials and Methods, Risk of bias and certainty of evidence; Certainty of Evidence |
| Results | 16 | Describe results of the search and selection process. | Results, Study selection and evidence classification; Figure 1 |
| Results | 17 | Cite each included study and present its characteristics. | Results; Tables 1 and 2; References |
| Results | 18 | Present assessments of risk of bias for included studies. | Results, Risk-of-bias assessment; Supporting Information Table S2 |
| Results | 19 | Present results of individual studies and syntheses. | Results, Primary meta-analysis; Table 2; Figure 2 |
| Results | 20 | Present results of all statistical syntheses conducted. | Results, Primary meta-analysis; Secondary exploratory analysis |
| Results | 21 | Present assessments of risk of bias due to missing results where available. | Materials and Methods; Discussion limitations |
| Results | 22 | Present assessments of certainty of evidence. | Certainty of Evidence; Table 3 |
| Discussion | 23 | Provide a general interpretation of results in context. | Discussion |
| Discussion | 24 | Discuss limitations of the evidence included in the review. | Discussion |
| Discussion | 25 | Discuss limitations of the review processes. | Discussion |
| Discussion | 26 | Discuss implications for practice, policy and future research. | Discussion; Conclusion |
| Other information | 27a | Provide registration information for the review. | Materials and Methods, Study design and reporting |
| Other information | 27b | Indicate where the protocol can be accessed, or state that none was registered. | Materials and Methods, Study design and reporting |
| Other information | 27c | Describe and explain amendments to information provided at registration or in the protocol. | Materials and Methods, Study design and reporting |
| Other information | 28 | Describe sources of financial or non-financial support. | Title page, Funding Statement |
| Other information | 29 | Declare any competing interests. | Title page and main text, Conflict of Interest Statement |
| Other information | 30 | Report availability of data, code and other materials. | Data Availability; Supporting Information |
